# Dynamics of Changes in pH and the Contents of Free Sugars, Organic Acids and LAB in Button Mushrooms during Controlled Lactic Fermentation

**DOI:** 10.3390/foods11111553

**Published:** 2022-05-25

**Authors:** Ewa Jabłońska-Ryś, Aneta Sławińska, Katarzyna Skrzypczak, Karolina Goral

**Affiliations:** 1 Department of Plant Food Technology and Gastronomy, University of Life Sciences in Lublin, Skromna 8, 20-704 Lublin, Poland; aneta.slawinska@up.lublin.pl (A.S.); katarzyna.skrzypczak@up.lublin.pl (K.S.); 2 Clinical Dietetics Unit, Medical University of Lublin, Chodzki 7, 20-093 Lublin, Poland; karolina.goral@umlub.pl

**Keywords:** button mushroom, *Agaricus bisporus*, *Lactiplantibacillus plantarum* 299v, *Lactiplantibacillus plantarum* EK3, lactic fermentation, free sugars, organic acids

## Abstract

The aim of the study was to assess changes in the basic quality parameters induced by controlled lactic fermentation of fruiting bodies of the button mushroom (*Agaricus bisporus*). *Lactiplantibacillus plantarum* 299v with documented probiotic properties and *L. plantarum* EK3, i.e., an isolate obtained from spontaneously fermented button mushrooms, were used as starter strains. The fruiting bodies of fresh, blanched, and fermented mushrooms were analyzed at different stages of the lactic fermentation process. The content of free sugars (high-performance liquid chromatography with charged aerosol detector method; HPLC-CAD) and organic acids (high-performance liquid chromatography with diode array detector method; HPLC-DAD) was determined both in the mushroom fruiting bodies and in the brine. Five free sugars (ribose, trehalose, sucrose, glucose, and fructose), mannitol, and six organic acids (lactic, malic, succinic, citric, acetic, and fumaric acids) were detected in the samples. Lactic acid dominated in the final products. The starter cultures exhibited varying degrees of utilization of available mushroom sugars and sucrose used as an additional substrate. Sucrose was utilized at a higher rate and in greater amounts by the *L. plantarum* EK3 isolate. This starter culture was characterized by a significantly higher final amount of produced lactic acid, a lower pH value, and higher numbers of LAB (lactic acid bacteria). These important quality parameters largely determine the stability of fermented products. Based on the analysis results and the high scores in the organoleptic evaluation of the fermented mushrooms, the *L. plantarum* EK3 isolate can be recommended as an appropriate starter culture for lactic fermentation of mushroom fruiting bodies.

## 1. Introduction

Lactic fermentation-based preservation of food has a very long tradition. It is one of the oldest preservation methods widely used in many countries of the world, mainly in Africa and Asia and in Eastern European countries [1,2,3]. This method can be used to preserve raw materials of plant (vegetables, fruits, grains, herbs) and animal (milk, meat, fish) origin, as well as mushrooms [3,4,5,6]. The lactic fermentation process contributes to the extension of product shelf life, protection against the growth of undesirable microorganisms, improvement of nutritional values (enrichment with some vitamins, elimination of anti-nutritional compounds), increased digestibility, reduction of calorific value, and modification of the flavor of fermented raw materials [2,4,5].

Despite the availability of starter cultures, lactic fermentation is mostly a spontaneous process, especially in the case of raw materials of plant origin [1,7]. Sauerkraut, which is produced via spontaneous lactic fermentation regardless of the production scale, is a popular fermented product in Poland. The fermentation method has advantages, as it is cheap and, when carried out properly, yields a high-quality product with desired sensory properties [8,9]. However, initiation of the spontaneous fermentation process takes a relatively long time (24–48 h) and is associated with a high risk of failure [1]. The use of starters ensures prompt control of the fermentation environment by lactic bacteria protecting the product against the growth of unfavorable microflora, which is particularly important in the initial fermentation period. It also shortens the process and facilitates standardization of the finished product [7,9].

Mushrooms do not undergo spontaneous fermentation readily [6], due to the blanching process that has to be applied to this raw material. Therefore, the use of starter cultures is necessary in this case; both autochthonous microflora, isolated from fresh raw material or material subjected to spontaneous lactic fermentation, and allochthonous microflora, isolated from other sources, can be used as starters in the fermentation process [9,10]. Starter cultures composed of allochthonous microflora are becoming increasingly popular due to their potential probiotic properties [11].

The *Lactiplantibacillus plantarum* 299v strain (former name: *Lactobacillus plantarum* 299v) with documented probiotic properties is the best described *L. plantarum* strain worldwide. It was isolated from the healthy human intestinal mucosa [12]. Many studies have been conducted to evaluate the possibility of using this strain in the production of food of both plant and animal origin [13,14,15,16]. Our research team has evaluated the potential use of probiotic strain *L. plantarum* 299v in the process of lactic fermentation of button mushroom fruiting bodies [17].

To the best of our knowledge, there are no studies on the use of autochthonous microflora in fermentation of mushrooms. One of the isolates obtained previously by our research team through spontaneous fermentation of mushroom fruiting bodies (*Agaricus bisporus*) [18] was used in the present study.

It is essential in the process of controlled lactic fermentation that starter cultures utilize available carbohydrates for rapid production of large amounts of lactic acid. The dynamics of changes in the levels of these compounds is therefore an important indicator of the correct course of lactic fermentation. There is only one publication describing changes in the content of organic acids observed during lactic fermentation of mushrooms [19]. Two other papers demonstrated changes in the content of total sugars [20,21], but none of the studies were focused on fermentation of mushroom fruiting bodies. The present study investigated the dynamics of changes in the content of free sugars and organic acids during controlled lactic fermentation of button mushroom fruiting bodies. The button mushroom is the most popular species of edible mushrooms in Poland [22] and one of the four most popular fungal species in the world [23].

## 2. Materials and Methods

### 2.1. Chemicals, Reagents, and Standards

HPLC-grade acetonitrile 99.9% was obtained from Merck (Darmstadt, Germany). Other reagents: (i) ethanol 96% (analytical grade) was purchased from POCH (Gliwice, Poland), (ii) formic acid 98–100% (for liquid chromatography–mass spectrometry LiChropur^®^) was purchased from Sigma-Aldrich (Saint Louis, MO, USA), and (iii) standards (analytical grade; all contents > 99%) of sugars (ribose, fructose, glucose, sucrose, trehalose), sugar alcohol (mannitol), and organic acids (lactic, malic, succinic, citric, acetic, fumaric) were supplied by Sigma-Aldrich (Saint Louis, MO, USA). All aqueous solutions were prepared using ultra pure water from the water purification system Direct-Q 3UV (Merck Millipore, Darmstadt, Germany). 

### 2.2. Raw Materials

Fruiting bodies of the button mushroom *Agaricus bisporus* were the study material. They were purchased from a producer and intended for further processing immediately after harvest (maximum after 4 h). Two strains of lactic acid bacteria (LAB): *Lactiplantibacillus plantarum* 299v with documented probiotic properties (Probi AB, Lund, Sweden) used in a previous study [17] and *L. plantarum* EK3, i.e., one of the isolates obtained from fermented button mushrooms in previous research, were the starter cultures [18]. LAB were propagated twice in MRS broth (Biocorp, Warsaw, Poland) and incubated (TK-2, Cabrolab, Warsaw, Poland) overnight at 30 °C. After centrifugation (MPW 350-R, MPW, Warsaw, Poland) at 1400× *g* for 10 min, microbial cells were harvested and washed twice in sterile 0.9% NaCl (P.O.Ch., Gliwice, Poland) before inoculation.

### 2.3. Preparation of Fermented Mushrooms

The fermentation procedure was based on a previous study [17] with some modifications. Fruiting bodies with a diameter of 3.5–4.5 cm with stipes trimmed short were chosen for the fermentation process. The mushrooms were cleaned thoroughly to remove substratum debris, washed, and blanched in boiling water for 2 min. After cooling, 2% (*w*/*w*) NaCl and 1% (*w*/*w*) sucrose were added. The mushrooms were divided into portions of 380 g each, placed in 500 mL glass jars, and 120 mL of a solution containing 2% NaCl and 1% sucrose was added. Then, the starter cultures (probiotic *L. plantarum* 299v or *L. plantarum* EK3) were inoculated at 10^7^ CFU (colony-forming unit) per gram of the materials subjected to the fermentation process and the jars were closed. The initial pH of this food matrix was approximately 6.8 (Table 1). Lactic fermentation proceeded for 7 days at 21–22 °C, after which the fermented mushrooms were stored at 5 °C for 5 weeks for maturation. 

Brine and mushroom samples were collected for physicochemical and microbiological analyses on day 0, 2, 4, 7, 14, and 42 of the experiment. Additionally, on day 42, a sensory evaluation of the finished products was performed. Samples were stored at −80 °C until the determination of free sugars and organic acids, and the other analyses were performed on an ongoing basis. 

### 2.4. Determination of pH

The pH value was measured in the brine during fermentation using a digital pH meter (Seven Compact S210, Mettler Toledo, Greifensee, Switzerland). All measurements were performed in triplicate and expressed as a mean ± standard deviation (SD).

### 2.5. Microbiological Analysis 

The amount of LAB in the brine during fermentation was measured according to PN-ISO 15214:2002 [24]. The counts were expressed as the log of colony forming units (CFU) per milliliter of the sample. All measurements were performed in triplicate and expressed as a mean ± standard deviation (SD).

### 2.6. Determination of Free Sugars

#### 2.6.1. Extraction Procedure

Free sugars and the polyol were analyzed using a method described by Sławińska et al. [25]. Suspensions of 2 g of homogenized mushrooms or 2 mL of brine in 8 mL of 80% ethanol were shaken at 150 rpm for 0.5 h at 80 °C. After this time, the mixture was centrifuged for 15 min at 5000× *g* (MPV-350R, Warsaw, Poland). Then, 1 mL of the supernatant was taken and 3 mL of acetonitrile was added and placed in the freezer for 24 h. Next, the samples were centrifuged for 15 min at 16,000× *g* to obtain clear supernatants, which were used for subsequent HPLC-CAD analyses. All assays were carried out in triplicate.

#### 2.6.2. Instrumentation and Chromatographic Conditions

Free sugars and the polyol were analyzed using an Ultimate 3000 HPLC system coupled to a Corona-Veo RS-charged aerosol detector (Germering, Germany). The CAD detector parameters were set as follows: nitrogen gas pressure: 35 psi; detector response 100 pA; noise filter: high. In addition, the UV signal was registered at 210 nm by a Dionex Ultimate 3000 RS Diode Array Detector (Thermo Fisher Scientific Inc., Waltham, MA, USA). Separation was carried out using a Shodex Asahipak NH2P-50 4E 5 µm (4.6 × 250 mm) column (Showa Denko, Tokyo, Japan) with an Asahipak NHZP-506 4A precolumn (Showa Denko, Tokyo, Japan). The mobile phase was composed of 75% acetonitrile and 25% water. The flow rate was 1 mL/min. The column temperature was maintained at 30 °C. The injection volume was 20 µL. The chromatographic separation time was set to 22 min. Chromoleon Dionex Software version 7.2 SR4 was used for data acquisition, instrument control, and data analysis.

The sugars and sugar alcohol were identified by comparing the retention times of sample peaks with standards in the same chromatographic conditions. The quantification was carried out with the internal standard method. The results were expressed in mg per 100 g of mushroom fruiting bodies or 100 mL of brine. All measurements were expressed as a mean ± standard deviation (SD).

### 2.7. Determination of Organic Acids

#### 2.7.1. Extraction Procedure

Suspensions of 1 g of homogenized mushrooms or 1 mL of brine in 8 mL of a 0.1% solution of formic acid in deionized water were shaken at 150 rpm for 30 min at 25 °C. After this time, the mixture was centrifuged for 15 min at 16,000× *g* (MPV-350R, Warsaw, Poland). Then, the supernatant was filtered through a 0.45 µm micropore filter membrane to obtain clear supernatants, which were used for subsequent liquid chromatography with ultra-violet absorption detection (HPLC-UV) analyses. All assays were carried out in triplicate.

#### 2.7.2. Instrumentation and Chromatographic Conditions

Organic acids were determined on a Dionex Ultimate 3000 HPLC system (Thermo Fisher Scientific Inc., Waltham, MA, USA) equipped with a Dionex Ultimate 3000 RS diode array detector (Thermo Fisher Scientific Inc., Waltham, MA, USA). The UV-signal was registered at 210 nm. Separation was carried out on a Zorbax SB-Aq 1.8 µm (2.1 × 100 mm) column (Agilent Technologies, Santa Clara, CA, USA) at 30 °C. 0.1% formic acid in ultrapure (type I) water (Direct-Q 3UV, Merck Millipore; Burlington, VT, USA) was used as the mobile phase. The flow rate was 0.06 mL/min, and the chromatographic separation time was 12 min. The injection volume was 1 µL. Chromoleon Dionex Software version 7.2 SR4 (Thermo Fisher Scientific Inc., Waltham, MA, USA) was used for data acquisition, instrument control, and data analysis.

The identification of organic acids was performed by comparing the retention times of sample peaks with standards in the same chromatographic conditions. The quantification was carried out with the external standard method. The organic acid content was expressed in mg per 100 g of mushroom fruiting bodies or 100 mL of brine. All measurements were expressed as a mean ± standard deviation (SD).

### 2.8. Sensory Evaluation

A 5-point hedonic scale (5 = excellent, 4 = very good, 3 = good, 2 = bad, 1 = very bad) was employed for the evaluation of the sensory parameters of the fermented mushrooms. A panel of nine judges assessed the color, aroma, taste, texture, and overall quality of the finished products. All samples were coded with two random digit numbers, and the serving order was randomized as well. The results were expressed as mean value ± standard deviation (SD).

### 2.9. Statistical Analysis

Statistical analysis was performed using the STATISTICA 13.1 program (StatSoft, Cracow, Poland), with Tukey’s HSD test in the analysis of variance (ANOVA) to estimate the significance of the differences between the mean values at *p* < 0.05

## 3. Results and Discussion

### 3.1. Changes in the pH Value during Mushroom Fermentation

The pH value is a very important indicator of fermentation progress, and its decrease is associated with the presence of organic acids, mainly lactic acid released by LAB into the medium [19]. As shown in Table 1, the initial pH value was lower than 6.8 in both starter variants and declined sharply on the first fermentation days. On day 4 of the experiment, the pH dropped below 4 in both cases and reached a stable value after 7 days of fermentation, which was maintained until the end of the experiment. Initially, the rate of the changes in the pH value was independent of the strain used. However, on day 4, the differences between the mushroom samples fermented by the different starter cultures were statistically significant. The pH value in the fully fermented mushrooms was significantly lower in the *L. plantarum* EK3-fermented variant (3.55 ± 0.03) than in the mushrooms fermented by *L. plantarum* 299v (3.68 ± 0.03).

These results were similar to the findings reported in our previous studies [17] and those described by Skąpska et al. [26] in a study on mushrooms. The pH value in other fermented mushroom species has been reported to range from 3.3 to 4.6 and depends on, e.g., the fermentation temperature, the amount of available carbohydrates, or the additives used in the fermentation process [6]. As reported by Steinkraus [4], a pH value below 4.0 ensures stability of fermented vegetables with simultaneous maintenance of anaerobic conditions.

### 3.2. Microbiological Changes during Fermentation

The number of LAB introduced with the starter cultures was 7.11 ± 0.03 and 7.06 ± 0.04 log CFU/mL in the *L. plantarum* 299v and *L. plantarum* EK3 variants, respectively (Table 2). The probiotic LAB population increased rapidly and reached a maximum, i.e., 9.17 ± 0.07 log CFU/mL, on day 4 of the experiment. The isolate reached the maximum count of 9.05 ± 0.02 log CFU/mL on day 2. After the one-week fermentation period, the number of LAB gradually decreased and, finally in the finished product, was 7.11 ± 0.02 and 7.68 ± 0.04 log CFU/mL in the *L. plantarum* 299v- and *L. plantarum* EK3-fermented samples, respectively. In a study conducted by Skąpska [26], the maximum number of LAB (approx. 9 log CFU/mL) was recorded on fermentation day 4; in the finished product, the number declined to approximately 8 log CFU/mL. As reported by Liu et al. [19], the population of LAB in three kinds of fermented mushroom products increased rapidly to approx. 8.5 log cfu/mL during the first 3 days and then decreased to 7.5 log cfu/mL on day 18. LAB populations are reduced due to the inhibitory effect of low pH, the high concentration of organic acids, and the reduction of the amount of available carbohydrates [19].

Between day 7 and the end of the experiment, statistically significantly higher numbers of LAB were found in the isolate-fermented mushrooms than in the probiotic strain fermentation variant. This was reflected in the pH value in the fermented mushrooms, which was already significantly lower in the *L. plantarum* EK3 fermentation variant on day 4 (Table 1). 

### 3.3. Changes in the Levels of Free Sugars and Mannitol during Fermentation

The content of carbohydrates used as a carbon source by lactic acid bacteria in raw materials intended for fermentation is an important parameter. Free sugars were represented in the analyzed mushroom fruiting bodies by three monosaccharides (glucose, fructose, and ribose), two disaccharides (sucrose and trehalose), and one polyol (mannitol). Mannitol and ribose are the dominant sugars in fresh mushroom fruiting bodies; their levels were estimated at 738.43 ± 26.58 and 377.08 ± 33.73 mg/100 g of fresh mass, respectively. The content of trehalose, which is a characteristic mushroom disaccharide, was estimated at 116.46 ± 6.85 mg/100 g of fresh mass. The amounts of sucrose, glucose, and fructose were substantially smaller (Table 3). The available literature data in this field vary. However, studies conducted by other authors have evidenced that mannitol is the main component of the soluble carbohydrate fraction in *Agaricus bisporus* [27,28]. Together with free 5′-nucleotides and free amino acids, mannitol is the main component responsible for the taste of mushrooms [29].

The sugar content is influenced by many factors, e.g., the stage of maturity or the morphological part of the mushroom. These values also change during the post-harvest storage of mushrooms [27,28,29,30]. The mushrooms analyzed in the present study were harvested at an early stage of maturity. Following the five maturity categories proposed by Tsai et al. [27] (stage 1—pin head stage; 2—veil intact, tight; stage 3—veil intact stretched; stage 4—veil opened; stage 5—gills exposed), the mushrooms used in the present study represented stage 2 and were processed within 4 h after harvesting.

The blanching process reduced the content of the analyzed sugars. No glucose, fructose, or sucrose were detected in the blanched mushroom samples. The content of mannitol and trehalose decreased significantly to the level of 543.4 ± 47.6 and 93.51 ± 5.55 mg/100 g of fresh mass, respectively. The ribose content was reduced as well, but the decline was not statistically significant (Table 3). Probably, the losses of the analyzed compounds were associated with soluble sugar leaching into water. This was confirmed by Li et al. [31] in their study on the influence of water blanching and microwave blanching on the soluble sugar and polyol content in *Lentinula edodes*. As suggested by the authors, Maillard reactions occurring during high-temperature blanching may be another possible cause of sugar loss. Despite the negative impact of blanching on the sugar content, this procedure cannot be ignored. Its main aim is to remove air from mushroom tissues, which creates anaerobic conditions. This is essential for the proper course of the lactic fermentation process. Moreover, the blanching procedure inactivates enzymes and reduces microflora abundance [6].

The dynamics of changes in the sugar content induced by the fermentation process was investigated both in the mushroom fruiting bodies and in the brine. Samples for analyses were collected on experimental days 2, 4, 7, 14, and 42. The content of sugars underwent dynamic changes, which were correlated significantly with the starting culture used for the fermentation process.

The content of ribose in the mushrooms on day 2 of the fermentation process significantly decreased in comparison with its level in the blanched mushrooms (Table 3). It seems that the decline can mainly be explained by the process of leaching, as large amounts of this monosaccharide were present in the brine (Table 4). Throughout the fermentation period, the content of ribose in the *L. plantarum* 299v-fermented samples was similar both in the fruiting bodies and in the brine. The content of this compound in the *L. plantarum* EK3-fermented samples exhibited certain fluctuations. The content of ribose both in the fruiting bodies and in the brine increased on day 4 and then decreased again.

As shown by Westby et al. [32], *L. plantarum* strains differ in the utilization of ribose as a carbon source. Some strains do not grow on substrates supplemented with ribose as the only carbon source, whereas others are able to metabolize the compound. Most *L. plantarum* strains, however, require the presence of glucose and amino acids to induce the enzyme system for ribose metabolism. Ribose used in fermentation is metabolized into lactic and acetic acids. Our previous investigations have shown that the *L. plantarum* EK3 isolate can grow on a substrate with ribose as the only carbon source [18].

Fructose, glucose, and sucrose, which were not detected in the blanched mushrooms, were present in the fermented mushrooms and the brine (Table 3 and Table 4) due to the addition of 1% of sucrose as an ingredient in the recipe. Sugar is used as an easily available source of carbon for lactic bacteria. Most authors of available studies on fermented mushrooms recommend the addition of sucrose, most often in the amount of 1% [6]. Instead of pure sucrose, table sugar, which may contain some glucose and fructose, was used in the study [33,34]. The small amounts of these simple sugars in the fermented mushroom samples and the brine may therefore originate from the added table sugar or from sucrose hydrolysis [34]. The presence of glucose and fructose in the fruiting bodies and the brine was detected only in the isolate-fermented samples (Table 3 and Table 4). The amounts of these sugars declined substantially along the fermentation process. As demonstrated by Yang et al. [35], LAB prefer reducing sugars, mainly glucose and fructose, as a carbon source. However, due to their low content in mushroom fruiting bodies, these sugars cannot play a key role in the lactic fermentation process, unlike in the fermentation of cabbage. As reported by Xiong et al. [34], even if glucose is present in the raw material, the addition of sucrose significantly reduces the utilization thereof. The authors revealed an even lower degree of utilization of fructose, whose amount in the finished fermented product (sauerkraut) was significantly higher than the initial content, i.e., greater amounts of this sugar were generated by, e.g., sucrose transformation than the amount consumed by LAB. Milanovic et al. [36] used an addition of sucrose, glucose, or fructose as a medium for lactic bacteria. The authors observed the fastest decrease in the pH value in samples of fermented fungi supplemented with glucose. Nevertheless, after 10 days of fermentation, the lactic acid content was similar in all samples, regardless of the type of sugar added. 

In the initial period of fermentation, sucrose was mainly present in the brine. The concentration of this compound immediately after the start of the fermentation process was over 3.3 g/100 mL in the brine and slightly over 200 mg/100 g in the mushroom fruiting bodies (see Figure S1 in the Supplementary Materials), as the sugar had not yet saturated the raw material tissues. On day 2 of the experiment, the greatest decrease in the sucrose content in the brine and an increase in the mushroom tissues were observed. Throughout the fermentation period, water-soluble components, such as sugars and acids, migrate between the brine and the raw material, and the sugars are gradually utilized by LAB to produce organic acids (mainly lactic acid). The sucrose concentration in the raw material and the brine on day 14 was identical due to the saturation of mushroom tissue with this sugar on the one hand and the utilization thereof from the brine by LAB on the other hand (Table 3 and Table 4). In a study on fermented cabbage conducted by Xiong et al. [34], equal concentrations of sucrose in brine and shredded cabbage were noted after 72 h of fermentation. However, the cabbage was finely shredded, whereas whole mushrooms were used in the present study. The degree of fragmentation of the raw material had a significant effect on the rate of concentration equalization. 

The largest decrease in sugar content in the first fermentation period is closely associated with the intensive growth of LAB in an anaerobic environment (Table 2) and production of high amounts of lactic acid (Table 5 and Table 6), which is discussed in greater detail in Section 3.4.

It was observed that sucrose was not completely utilized by LAB in the fermentation process. Significantly lower amounts of this sugar were left in the *L. plantarum* EK3 isolate fermentation variant (132.42 ± 1.4 mg/100 g of mushrooms and 134.27 ± 1.78 mg/100 mL of brine) than in the probiotic strain variant (271.45 ± 2.25 mg/100 g of mushrooms and 261.05 ± 3.94 mg/100 mL of brine). This may indicate that the isolate exhibited a higher sucrose utilization rate and produced greater amounts of lactic acid. Possibly, the 1% addition of sucrose suggested by most authors to be used in the mushroom fermentation process [6] is too high. However, even greater amounts of sucrose (2–3%) were used in some studies [19,36,37,38]. 

The content of trehalose in the fermented mushrooms was significantly lower than in the blanched material. Very similar trehalose contents were detected in the brine. The mushrooms and the brine exhibited equal levels of this compound (Table 3 and Table 4). The content of this sugar in the mushroom fruiting bodies and the brine was significantly lower in the samples fermented with the probiotic strain, which may indicate greater efficiency of *L. plantarum* 299v in utilization of trehalose in comparison with *L. plantarum* EK3. Our previous research demonstrated the ability of the *L. plantarum* EK3 isolate to grow on a trehalose-supplemented substrate [18]. Trehalose is a disaccharide used as a storage compound by some microbes. It is an important source of carbon for lactic acid bacteria supporting their growth and increasing the production of bacteriocins [39]. Trehalose also plays an important role in enhancement of the tolerance of lactic acid bacteria to acidic environments, thereby influencing their viability at pH 3 or at even lower values [40].

There were no significant differences in the mannitol content in the fermented versus blanched mushrooms (Table 3). A significant decline in the content of this compound in the fruiting bodies and the brine was found only in the probiotic strain-fermentation variant on day 42 (Table 3 and Table 4). Interestingly, mannitol leached into the brine relatively quickly, and its concentrations in the mushroom fruiting bodies and in the brine were similar already on day 2. Concurrently, there was no significant decrease in the content of this compound, compared to that in the blanched mushrooms. Mannitol is a polyol that can be used as a carbon source. Both *L. plantarum* 299v and *L. plantarum* EK3 were able to grow on a mannitol-supplemented medium [18,41]. On the other hand, this compound can also be produced by lactic acid bacteria in the heterofermentation process. Mannitol can be converted from glucose, fructose, and sucrose mainly by heterofermentative bacteria. Some homofermentative LAB produce some amounts of this compound as well [42]. The most probable metabolic pathway for mannitol production is the reversion of mannitol catabolism via mannitol-1-phosphate (mannitol-1P) dehydrogenase, an enzyme whose activity has been detected in *L. plantarum* [43]. In contrast to mannitol production, its utilization is more common among homofermentative lactic acid bacteria [42].

Unfortunately, the present results cannot be compared with any other findings in this field. To our knowledge, no similar research on mushrooms has been carried out to date. There are only two studies assessing the impact of the fermentation process on the total sugar content in fermented mushrooms. As reported by Ogidi and Agbaje [20], the carbohydrate content in the lactic fermentation process decreased from 77.4% of dry mass of fresh *Pleurotus ostreatus* fruiting bodies to 67.3% of dry mass in fermented samples. In turn, as reported by Choi et al. [21], the content of total free sugars decreased from 9.68 mg% in aqueous extracts of *Lentinula edodes* fruiting bodies to 8.19–9.31 mg% in fermented extracts, depending on the strain used. In both studies, 3% sucrose [20] or 3% lactose and 2% sucrose [21] were added to the bacterial growth medium. Also, other fungal carbohydrates, e.g., polysaccharides (glucans), glycogen, and chitin, can be used as an energy source for LAB [20].

### 3.4. Changes in the Levels of Organic Acids during Fermentation

Six organic acids were identified in the fresh mushroom fruiting bodies (Table 5), with the greatest amounts of malic acid and succinic acid (476.18 ± 21.42 and 465.6 ± 84.92 mg/100 g, respectively), followed by lactic acid and citric acid (280.59 ± 19.6 and 273.23 ± 26.52 mg/100 g, respectively), and the lowest contents of fumaric and acetic acids (40.02 ± 0.92 and 67.55 ± 1.62 mg/100 g, respectively). The qualitative and quantitative composition of organic acids in *A. bisporus* fruiting bodies may vary largely depending mainly on the strain. Gąsecka et al. [44] analyzed the organic acid profile in fruiting bodies of various strains of cultivated button mushrooms. Succinic acid (2242.2–11478.4 mg/100 g of dry mass) was the dominant acid in five of the six analyzed strains. Similarly, the button mushrooms analyzed by Pei et al. [45] exhibited the predominance of succinic acid. In turn, Barros et al. [46] showed the highest amounts of citric and malic acids, i.e., 43.23 and 29.51 mg/g of dry mass, respectively, in button mushroom fruiting bodies. As demonstrated by Aisal et al. [47], malic acid clearly dominated among the organic acids present in mushroom fruiting bodies.

Organic acids have an impact on the taste and aroma of mushrooms, especially malic [46] and succinic [31] acids. These compounds also play an important biological role. They have antioxidant properties, e.g., malic, citric, and succinic acids [46] prevent the enzymatic darkening process, which is a considerable problem during harvesting, marketing, storage, and processing of button mushrooms [22,48]. Organic acids play a particularly important role in products subjected to lactic acid fermentation, as they largely determine the stability of the finished product [4,9,11]. Their antibacterial and antifungal properties contribute to inhibition of the growth of undesirable microflora in fermented products [44,49].

The blanching process applied in the present study resulted in a decrease in the content of organic acids (Table 5), mostly succinic acid, whose content exhibited an almost 10-fold decrease. In a study conducted by Li et al. [31], the blanching process resulted in a 100% loss of this compound. As explained by the authors, this was caused by the decarboxylation process accompanying the hot water blanching procedure. 

Malic acid, which dominated in the fresh mushrooms, was present in the blanched mushrooms by a four-fold lower amount. With the exception of lactic acid, the levels of all the other acids were statistically significantly reduced, probably due to the process of leaching into the water or the high temperature in the case of volatile acids, e.g., acetic acid.

The changes in the content of the analyzed acids induced by the fermentation process varied (Table 5 and Table 6). Lactic acid was the main acid produced by both strains during fermentation. *L. plantarum* is a homofermentative LAB metabolizing hexoses via the Embden–Meyerhof pathway, with pyruvate as the central branching point of metabolism. Further changes depend on the availability of oxygen and nutrients. Depending on the fermentation conditions, they may be facultatively heterofermentative; however, lactic acid is the predominant end product [11]. 

The content of lactic acid systematically increased during the fermentation process, with the greatest increase recorded in the initial period and it was accompanied by the highest decrease in the sucrose content observed during the experiment (Figure S1). The coefficient of correlation between the content of these compounds in the brine during the fermentation process and their content during the cold storage period was −0.974 and −0.957 in the *L. plantarum* 299v strain and EK3 isolate variants, respectively. Finally, its content was significantly higher in the *L. plantarum* EK3-fermented samples, i.e., 1531.28 ± 103.9 mg/100 g and 1520.91 ± 28.06 mg/100 mL in the mushrooms and the brine, respectively. In turn, the content of lactic acid in the probiotic strain-fermented samples was 1266.93 ± 35.37 mg/100 g and 1364.02 ± 10.09 mg/100 mL in the mushrooms and the brine, respectively. These results are consistent with the observations of the changes in the pH value in the fermented mushrooms, where the value of this parameter in the final product was significantly lower in the EK3 starter-fermented samples (Table 1). Sucrose, which was the main source of carbon in the lactic fermentation process, was utilized at a higher rate and in greater amounts by the *L. plantarum* EK3 isolate (Table 3 and Table 4). This starter was also characterized by a significantly higher LAB count (Table 2). 

The content of malic acid in the fermented mushrooms was significantly lower than in the blanched samples. This can be partially explained by the leaching of this compound from the mushrooms into the brine. However, a further decline in its content was observed in the brine along the fermentation process (Table 6). A decrease in the malic acid content during lactic fermentation of Pleurotus spp. fruiting bodies was reported by Liu et al. [19]. It may have been caused mainly by decarboxylation of malic acid to lactic acid by the malolactic enzyme produced by most LAB species [50]. This process is characteristic of malolactic fermentation, which is widely used for partial deacidification of wines [51]. Decarboxylation of malic acid yielding lactic acid and CO_2_ was also observed by McDonald et al. [52] in the process of fermentation of cucumbers by naturally occurring *L. plantarum* strains. The authors also report that some LAB strains can degrade 100% of malic acid. In turn, Tkacz et al. [53] indicate the greatest reduction of malic acid within the first 24–72 h of fermentation by *L. plantarum* strains, which is fully consistent with our observations.

The content of succinic acid in the initial fermentation stage was almost three-fold higher than that in the blanched mushrooms (Table 5). Similar amounts of this compound were also detected in the brine (Table 6). Similarly, Liu et al. [19] observed an increase in succinic acid content during lactic fermentation of *Pleurotus* spp. Succinic acid is produced via conversion of citric acid in the tricarboxylic acid cycle [50,54]. Kaneuchi et al. [55] reported that a number of *Lactiplantibacillus* strains produced various amounts of succinic acid in MRS broth. In the absence of oxaloacetate decarboxylase, the utilization of citrate proceeds through the succinic acid pathway. In such a case, lactate, acetate, and succinate are formed from glucose and citrate [56]. Succinic and malic acids are responsible for the aroma of fermented products [35]. The content of both of these acids in the final products was significantly higher in the probiotic strain-fermented samples.

During the fermentation process, the content of citric acid was significantly reduced in both the mushroom fruiting bodies and the brine, regardless of the starter culture used (Table 5 and Table 6). The amount of this acid on fermentation day 2 was similar (in the case of the mushroom fruiting bodies) or slightly higher (in the brine) to its amount in the blanched mushrooms. It systematically decreased from fermentation day 4. Similar findings (an initial slight increase followed by a decrease in the content of the acid) were reported by Liu et al. [19], who carried out fermentation of *Pleurotus* spp. This can be explained by the gradual degradation of citric acid by lactic acid bacteria. As shown by Mirmohammadi et al. [57], *L. plantarum* are able to metabolize citric acid as a carbon source during fermentation. C4 aromatic compounds may be the end product of these transformations. Tkacz et al. [53] reported that, probably due to the low pH value, *L. plantarum* revealed a higher preference for acids than sugars as a carbon source. On the other hand, as indicated before, citric acid can be converted to succinic [50] and acetic acids [54].

The amount of acetic acid systematically increased during the fermentation process. On day 42 of the experiment, its content in the probiotic strain-fermented samples was 90.72 ± 1.5 mg/100 g and 86.86 ± 5.31 mg/100 mL in the fruiting bodies and the brine, respectively. In the EK3 isolate fermentation variant, the fruiting bodies and the brine contained 76.35 ± 6.73 mg/100 g and 109.48 ± 4.49 mg/100 mL of acetic acid, respectively. Similar results were reported by Liu et al. [19]. As shown by Tkacz et al. [53], *L. plantarum* is one of the facultatively heterofermentative bacteria (heterolactic metabolism), which means that they are capable of producing lactic acid and other compounds, e.g., acetic acid, depending on the fermentation conditions. Acetic acid can also be formed from citric acid [53,54,58]. The presence of acetic acid in products fermented by *Lactiplantibacillus* strains may result from lactic acid degradation [56]. Acetic acid in combination with ethanol (heterofermentative metabolites) forms ethyl acetate, which improves the organoleptic properties of fermented products [35].

The content of fumaric acid in the initial fermentation period decreased significantly in relation to the amount detected in the blanched mushrooms. From day 4, the level of this acid was very low and remained practically unchanged (Table 5 and Table 6). Its content in the final product slightly exceeded 3 mg/100 g of the mushroom fruiting bodies or in 100 mL of the brine, regardless of the starting culture used. The low content of this acid is a positive phenomenon, as its higher concentrations may inhibit the growth of LAB [59,60].

### 3.5. Sensory Evaluation of Fermented Mushrooms

The results of the sensory evaluation of the fermented button mushrooms are presented in Table 7. Regardless of the starter culture used, the texture of the final product achieved the highest score, i.e., 4.56 points. In their comments, the panelists emphasized that the consistency of the fermented mushrooms resembled that of pickled mushrooms, but the former had a more delicate taste and a fuller aroma with a noticeable mushroom flavor. The marinade in pickled products is made of acetic acid, which gives them the sharp and strong taste. The color was a highly rated parameter as well (4 and 4.11 for the *L. plantarum* 299v and *L. plantarum* EK3 variants, respectively). The final product had an attractive creamy color. The isolate-fermented mushrooms achieved a higher score for the taste, aroma, and overall quality. With the exception of the score for the aroma, the differences related to the starter used were not statistically significant. It should be emphasized that the recipe in this experiment did not include aromatic-seasoning ingredients. The inclusion of these additives would probably have yielded a higher organoleptic evaluation score, as demonstrated in previous studies [17].

## 4. Conclusions

The use of different starters in the lactic fermentation process had a significant impact on the dynamics of changes taking place during this process. The study showed that the starter cultures utilized available sugars to a varying extent. The sucrose added in the technological process was not utilized completely by LAB, but its substantially lower content was finally found in the EK3 isolate-fermented mushroom and brine samples. In the future, reduction of the dose of this sugar may be taken into consideration. Sucrose residues can constitute an easily accessible nutrient substrate for yeasts and molds, which can grow at low pH and pose a potential threat to the quality of the fermented products. On the other hand, the presence of this sugar has an impact on the organoleptic properties of the final products, as it balances the sour flavor.

In the probiotic strain-fermented variant, the final product had significantly lower contents of ribose, trehalose, and mannitol than in the EK3 isolate variant. This may prove the higher efficiency of *L. plantarum* 299v in utilization of typical fungal sugars during lactic acid fermentation. However, the other parameters indicate better technological properties of the *L. plantarum* EK3 isolate. From day 7 of the experiment, statistically significantly higher numbers of lactic acid bacteria were detected in the *L. plantarum* EK3-fermented mushrooms than in the probiotic fermentation variant. This was also correlated with higher sucrose utilization efficiency, a greater increase in the content of lactic acid, i.e., the main determinant of the stability of fermented products, and a greater decrease in the pH value in the final product.

The *L. plantarum* EK3-fermented mushrooms achieved slightly higher scores in the overall organoleptic evaluation and significantly higher scores for the aroma. To improve the organoleptic properties, future research should investigate the use of complex starter cultures containing heterofermentative bacteria, e.g., *Leuconostoc mesenteroides*. These bacteria secrete metabolites that can exert a greater impact on the taste and aroma of the final product.

The use of starter cultures with known properties, dedicated to the specific mushroom raw material, will certainly improve the quality and stability of these fermented products. 

## Figures and Tables

**Table 1:** Evolution of pH values in fermented mushrooms.

Starter	LF0 ^1^	LF2	LF4	LF7	LF14	LF42
299v	6.79 ± 0.03 dA ^2^	4.31 ± 0.04 cA	3.99 ± 0.01 bA	3.68 ± 0.03 aA	3.66 ± 0.03 aA	3.68 ± 0.03 aA
EK3	6.78 ± 0.05 dA	4.32 ± 0.03 cA	3.92 ± 0.03 bB	3.57 ± 0.04 aB	3.53 ± 0.03 aB	3.55 ± 0.03 aB

^1^ LF—lacto-fermented samples on days 0, 2, 4, 7, 14, and 42 of the experiment. ^2^ Data represent mean ± SD of three replicates. Different lowercase letters in the same row and different capital letters in the same column indicate significant differences between mean values (*p* < 0.05).

**Table 2:** Evolution of the LAB number in fermented mushrooms (log CFU/mL).

Starter	LF0 ^1^	LF2	LF4	LF7	LF14	LF42
299v	7.11 ± 0.03 aA ^2^	9.07 ± 0.04 dA	9.17 ± 0.07 dB	7.96 ± 0.01 cA	7.58 ± 0.03 bA	7.11 ± 0.02 aA
EK3	7.06 ± 0.04 aA	9.05 ± 0.02 eA	8.59 ± 0.03 dA	8.63 ± 0.03 dB	7.89 ± 0.04 cB	7.68 ± 0.04 bB

^1^ LF—lacto-fermented samples on days 0, 2, 4, 7, 14, and 42 of the experiment. ^2^ Data represent mean ± SD of three replicates. Different lowercase letters in the same row and different capital letters in the same column indicate significant differences between mean values (*p* < 0.05).

**Table 3:** Content of free sugars and mannitol in mushrooms (mg/100 g).

**Starter**	**F ^1^**	**B**	**LF2**	**LF4**	**LF7**	**LF14**	**LF42**
	Ribose						
299v	377.08 ± 33.73 b ^2^	338.72 ± 12.55 b	254.51 ± 5.42 aB	260.2 ± 2.67 aA	235.58 ± 7.15 aA	232.12 ± 8.37 aA	225.65 ±3.71 aA
EK3	377.08 ± 33.73 c	338.72 ± 12.55 c	226.7 ± 2.27 aA	285.73 ± 7.7 bB	272.88 ± 19.53 abB	244.62 ± 11.62 abA	244.29 ± 10.22 abB
	Fructose						
299v	8.74 ± 0.85	ND ^3^	ND	ND	ND	ND	ND
EK3	8.74 ± 0.85 c	ND	12.2 ± 0.24 d	7.49 ± 0.1 b	9.85 ± 0.37 c	5.45 ± 0.19 a	5.24 ± 0.66 a
				Glucose			
299v	9.72 ± 0.52	ND	ND	ND	ND	ND	ND
EK3	9.72 ± 0.52 d	ND	7.71 ± 0.49 c	6.82 ± 0.4 c	4.36 ± 0.73 b	3.78 ± 0.9 b	2.23 ± 0.27 a
				Sucrose			
299v	53.5 ± 2.32 a	ND	395.93 ± 6.15 dB	318.97 ± 2.45 cB	311.04 ± 5.29 cB	277.45 ± 1.82 bB	271.45 ± 2.25 bB
EK3	53.5 ± 2.32 a	ND	365.36 ± 3.41 eA	277.69 ± 10.67 dA	264 ± 7.12 dA	205.65 ± 4.93 cA	132.42 ± 1.4 bA
				Trehalose			
299v	116.46 ± 6.85 c	93.51 ± 5.55 b	43.86 ± 0.75 aA	39.58 ± 0.59 aA	35.29 ± 0.88 aA	38.53 ± 0.48 aA	42.36 ± 0.71 aA
EK3	116.46 ± 6.85 c	93.51 ± 5.55 b	63.38 ± 0.36 aB	54.57 ± 3.81 aB	62.19 ± 2.4 aB	63.55 ± 1.61 aB	61.58 ± 0.98 aB
	Mannitol						
299v	738.43 ± 26.58 c	543.4 ± 47.6 b	542.16 ± 3.4 bA	535.02 ± 1.6 bA	548.25 ± 8.57 bB	510.4 ± 1.54 abA	454.09 ± 4.68 aA
EK3	738.43 ± 26.58 c	543.4 ± 47.6 ab	587.91 ± 2.1 bB	553.29 ± 13.26 abA	515.68 ± 18.16 aA	516.17 ±14.67 aA	513.83 ± 7.55 aB

^1^ F—fresh sample; B—blanched sample; LF—lacto-fermented samples on days 2, 4, 7, 14, and 42 of the experiment. ^2^ Data represent mean ± SD of three replicates. Different lowercase letters in the same row and different capital letters in the same column indicate significant differences between mean values (*p* < 0.05). ^3^ ND—not detected.

**Table 4:** Content of free sugars and mannitol in brine (mg/100 mL).

**Starter**	**LF2 ^1^**	**LF4**	**LF7**	**LF14**	**LF42**
	Ribose				
299v	284.8 ± 5.82 aB ^2^	284.6 ± 6.6 aA	269.97 ± 5.69 aA	266.21 ± 7.84 aA	286.48 ± 20.03 aB
EK3	272.27 ± 3.67 bA	356.67 ± 5.98 dB	318.44 ± 5.13 cB	323.03 ± 4.45 cB	188.29 ± 1.3 aA
	Fructose				
299v	ND ^3^	ND	ND	ND	ND
EK3	10.38 ± 0.31 c	8.49 ± 0.48 b	7.83 ± 0.29 b	7.53 ± 0.3 b	5.79 ± 0.75 a
	Glucose				
299v	ND	ND	ND	ND	ND
EK3	6.87 ± 0.07 c	4.25 ± 1.42 b	2.56 ± 0.51 ab	2 ± 0.91 a	2.29 ± 0.31 ab
	Sucrose				
299v	405.08 ± 6.96 dA	350.19 ± 4.22 cA	357.34 ± 7.08 cB	287.86 ± 11.64 bB	261.05 ± 3.94 aB
EK3	383.83 ± 14.11 eA	361.15 ±2.92 dB	292.94 ± 8.61 cA	205.82 ± 2.44 bA	134.27 ± 1.78 aA
	Trehalose				
299v	41.12 ± 0.65 abA	40.63 ± 0.69 aA	40.91 ± 0.69 aA	40.24 ± 1.92 aA	43.85 ± 0.72 bA
EK3	63.55 ± 4.74 aB	63.45 ± 0.89 aB	58.81 ± 0.98 aB	64.92 ± 1.4 aB	62.6 ± 0.86 aB
	Mannitol				
299v	544.67 ± 11.11 bA	549.17 ± 4.55 bA	640.3 ± 9.05 cB	537.14 ± 19.88 bA	469.88 ± 2.45 aA
EK3	585.88 ± 15.1 bB	665.59 ± 9.68 cB	602.61 ± 16.39 bA	523.9 ± 7.56 aA	541.89 ± 7.1 aB

^1^ LF—lacto-fermented samples on days 2, 4, 7, 14, and 42 of the experiment. ^2^ Data represent mean ± SD of three replicates. Different lowercase letters in the same row and different capital letters in the same column indicate significant differences between mean values (*p* < 0.05). ^3^ ND—not detected.

**Table 5:** Organic acid content in mushrooms (mg/100 g).

**Starter**	**F ^1^**	**B**	**LF2**	**LF4**	**LF7**	**LF14**	**LF42**
	Lactic Acid						
299v	280.59 ± 19.6 a ^2^	207.29 ± 26.36 a	765.72 ± 19.93 bA	1012.67± 80.3 cA	1138.86 ± 128.13 cdA	1198.72 ± 27.87 dA	1266.93 ± 35.37 dA
EK3	280.59 ± 19.6 a	207.29 ± 26.36 a	709.62 ± 29.31 bA	1074.81 ± 19.88 cA	1280.15 ± 14.66 dA	1348.44 ± 6.66 dB	1531.28 ± 103.9 eB
	Malic acid						
299v	476.18 ± 21.42 c	119.32 ± 7.89 b	26 ± 1.26 aA	19.38 ± 1.17 aA	18.71 ± 3.27 aA	15.94 ± 1.21 aB	19.46 ± 1.09 aB
EK3	476.18 ± 21.42 c	119.32 ± 7.89 b	35.25 ± 1.06 aB	18.47 ± 0.38 aA	18.53 ± 1.04 aA	13.21 ± 0.47 aA	11.37 ± 3.52 aA
				Succinic acid			
299v	465.6 ± 84.92 c	50.06 ± 7.26 a	143.46 ± 8.25 bA	129.17 ± 9.82 bA	115.09 ± 11.81 bA	132.24 ± 2.61 bB	136.47 ± 4.07 bB
EK3	465.6 ± 84.92 c	50.06 ± 7.26 a	143.41 ± 5.89 bA	133.3 ± 3.95 bA	127.43 ± 0.58 bA	122.97 ± 0.42 bA	112.23 ± 6.95 bA
				Citric acid			
299v	273.23 ± 26.52 d	221.39 ± 11.78 cd	228.28 ± 20.85 cdA	181.44 ± 42.03 bcA	125.87 ± 55.78 abA	126.5 ± 17.31 abA	62.86 ± 8.85 aA
EK3	273.23 ± 26.52 f	221.39 ± 11.78 de	230.35 ± 19.03 efA	179.21 ± 10.71 cdA	137.8 ± 12.38 bcA	99.18 ± 9.27 abA	78.19 ± 26.11 aA
	Acetic acid						
299v	67.55 ± 1.62 c	40.49 ± 0.94 a	56 ± 6.31 bA	76.37 ± 2.09 cdB	83.6 ± 3.95 deB	83.45 ± 2.41 deB	90.72 ± 1.5 eB
EK3	67.55 ± 1.62 bc	40.49 ± 0.94 a	40.61 ± 9.6 aA	55.5 ± 9.35 abA	63.98 ± 4.73 bcA	60.2 ± 5.8 bcA	76.35 ± 6.73 cA
				Fumaric acid			
299v	40.02 ± 0.92 d	31.23 ± 1.72 c	7.83 ± 0.18 bB	3.89 ± 0.31 aA	3.32 ± 0.4 aA	3.47 ± 0.11 aA	3.33 ± 0.14 aA
EK3	40.02 ± 0.92 d	31.23 ± 1.72 c	7.34 ± 0.23 bA	3.43 ± 0.1 aA	3.34 ± 0.03 aA	3.32 ± 0.03 aA	3.25 ± 0.27 aA

^1^ F—fresh sample; B—blanched sample; LF—lacto-fermented samples on days 2, 4, 7, 14, and 42 of the experiment. ^2^ Data represent mean ± SD of three replicates. Different lowercase letters in the same row and different capital letters in the same column indicate significant differences between mean values (*p* < 0.05).

**Table 6:** Organic acid content in brine (mg/100 mL).

**Starter**	**LF2 ^1^**	**LF4**	**LF7**	**LF14**	**LF42**
	Lactic Acid				
299v	974.65 ± 3.71 aA ^2^	1183.45 ± 46.49 bA	1282.92 ± 92.29 bcA	1257.5 ± 21.66 bcA	1364.02 ± 10.09 cA
EK3	977.37 ± 30.14 aA	1255.3 ± 7.59 bA	1402.79 ± 64.45 cA	1508.53 ± 28.83 dB	1520.91 ± 28.06 dB
	Malic acid				
299v	20.43 ± 1.07 cA	18.42 ± 1.28 bcA	18.61 ± 0.26 bcB	12.82 ± 0.88 aA	16.57 ± 1.18 bB
EK3	19.32 ± 1.27 bA	19.7 ± 0.96 bA	18.01 ± 0.18 bA	13.48 ± 1.36 aA	12.6 ± 1.25 aA
	Succinic acid				
299v	159.27 ± 4.81 dB	141.48 ± 5.42 bcA	124.91 ± 8.27 aA	129.1 ± 0.78 abA	150.19 ± 4.35 cdB
EK3	133.88 ± 2.99 bcA	139.99 ± 3.29 cA	125.64 ± 6.05 abA	142.77 ± 2.18 cB	117.55 ± 1.14 aA
	Citric acid				
299v	330.49 ± 28.56 dB	253.39 ± 26.75 cA	162.94 ± 11.24 bA	109.31 ± 7.84 aA	102.14 ± 13.11 aA
EK3	213.65 ± 26.77 bA	217.13 ± 16.38 bA	130.72 ± 26.45 aA	111.01 ± 5.14 aA	116.89 ± 3.44 aA
	Acetic acid				
299v	44.84 ± 6.13 aA	70.38 ± 6.2 bA	83.88 ± 2.04 bcA	89.64 ± 11.38 cA	86.86 ± 5.31 bcA
EK3	51.41 ± 11.61 aA	90.02 ± 6.8 bB	87.39 ± 7.93 bA	101.65 ± 10.36 bA	109.48 ± 4.49 bB
	Fumaric acid				
299v	8.22 ± 0.2 cB	3.9 ± 0.16 bB	3.45 ± 0.25 abA	3.21 ± 0.04 aA	3.42 ± 0.09 aA
EK3	4.65 ± 0.06 cA	3.6 ± 0.04 bA	3.22 ± 0.14 aA	3.64 ± 0.05 bB	3.3 ± 0.04 aA

^1^ LF—lacto-fermented samples on days 2, 4, 7, 14, and 42 of the experiment. ^2^ Data represent mean ± SD of three replicates. Different lowercase letters in the same row and different capital letters in the same column indicate significant differences between mean values (*p* < 0.05).

**Table 7:** Sensory evaluation of fermented mushrooms.

Starter	Color	Aroma	Taste	Texture	Overall Quality
299v	4 ± 0.5 a ^1^	3.33 ± 0.5 a	3.78 ± 0.67 a	4.56 ± 0.53 a	3.78 ± 0.44 a
EK3	4.11 ± 0.6 a	3.89 ± 0.6 b	4.11 ± 0.6 a	4.56 ± 0.53 a	4.11 ± 0.6 a

^1^ Data represent mean ± SD of nine replicates. Different lowercase letters in the same column indicate significant differences between mean values (*p* < 0.05).

## Data Availability

Not applicable.

## Supplementary Materials

The following supporting information can be downloaded at: https://www.mdpi.com/article/10.3390/foods11111553/s1, Figure S1: Changes in sucrose content in mushrooms and brine during fermentation and cold storage.
