# Supplementary figures and images for: Dynamics of Changes in pH and the Contents of Free Sugars, Organic Acids and LAB in Button Mushrooms during Controlled Lactic Fermentation
Source: Foods. 2022 May 25;11(11):1553. doi: 10.3390/foods11111553 (PMC9180291; doi:10.3390/foods11111553)

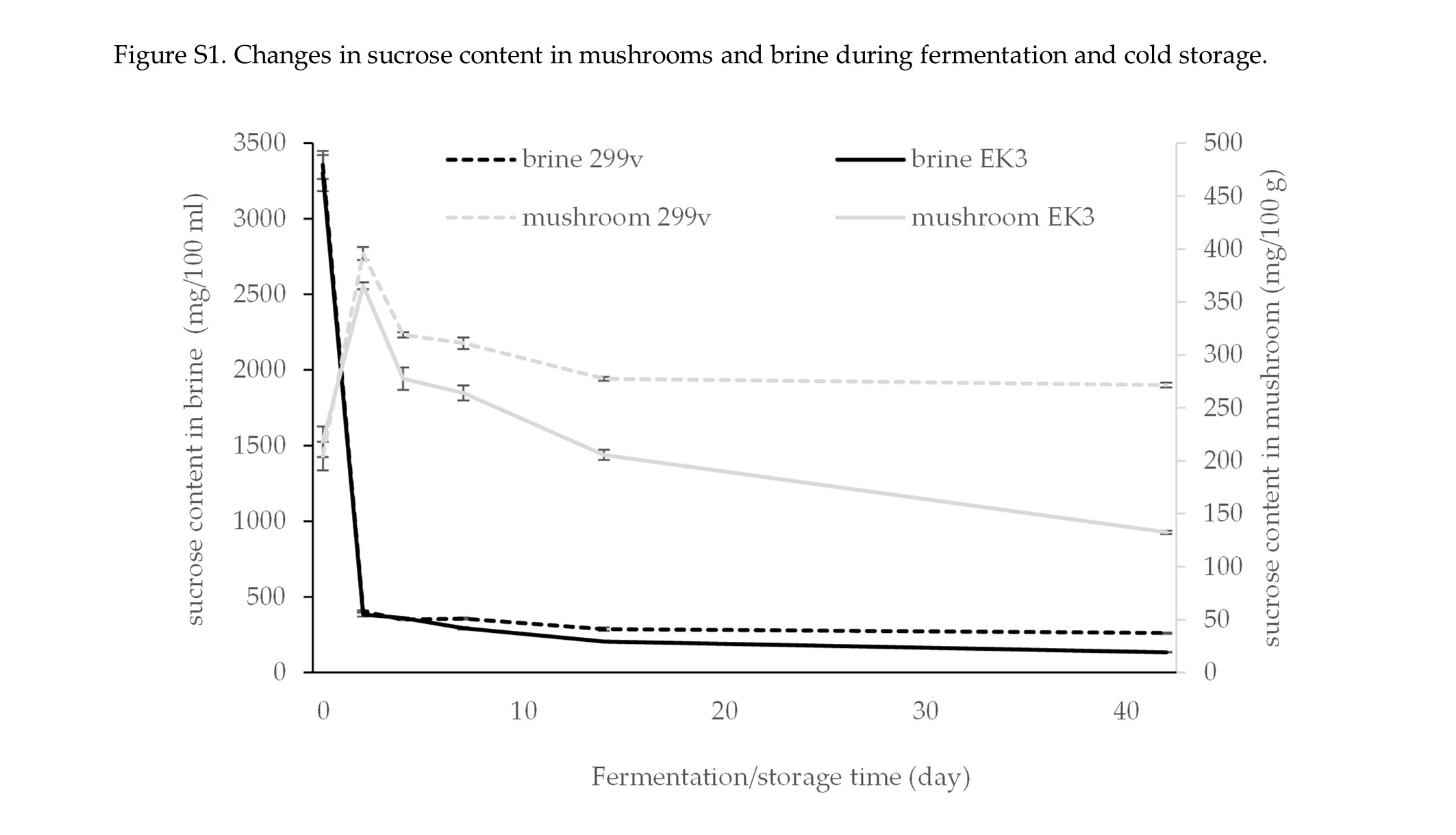

Supplement: Supplementary file 1 [file foods-11-01553-s001.zip › foods-1682154-supplementary.jpg]
